# Rhein-attenuates LPS-induced acute lung injury via targeting NFATc1/Trem2 axis

**DOI:** 10.1007/s00011-023-01746-8

**Published:** 2023-05-22

**Authors:** Xiang Li, Chuan Xiao, Jia Yuan, Xianjun Chen, Qing Li, Feng Shen

**Affiliations:** Department of Intensive Care Unit, The Affiliated Hospital of Guizhou Medical University, Guiyang, 550001 China

**Keywords:** Acute lung injury, Rhein, Macrophage polarization, Trem2, NFATc1

## Abstract

**Background:**

Evidence indicated that the early stage transition of macrophages’ polarization stages yielded a superior prognosis for acute lung injury (ALI) or acute respiratory distress syndrome (ARDS). Rhein (cassic acid) is one major component of many traditional Chinese medicines, and has been reported to perform with strong anti-inflammation capabilities. However, the role rhein played and the mechanism via which it did so in LPS-induced ALI/ARDS remain unclear.

**Methods:**

ALI/ARDS was induced by LPS (3 mg/kg, i.n, st), accompanied by the applications of rhein (50 and 100 mg/kg, i.p, qd), and a vehicle or NFATc1 inhibitor (10 mg/kg, i.p, qd) in vivo. Mice were sacrificed 48 h after modeling. Lung injury parameters, epithelial cell apoptosis, macrophage polarization, and oxidative stress were examined. In vitro, conditioned medium from alveolar epithelial cells stimulated by LPS was used for culturing a RAW264.7 cell line, along with rhein administrations (5 and 25 μM). RNA sequencing, molecule docking, biotin pull-down, ChIP-qPCR, and dual luciferase assay were performed to clarify the mechanisms of rhein in this pathological process.

**Results:**

Rhein significantly attenuated tissue inflammation and promoted macrophage M2 polarization transition in LPS-induced ALI/ARDS. In vitro, rhein alleviated the intracellular ROS level, the activation of P65, and thus the M1 polarization of macrophages. In terms of mechanism, rhein played its protective roles via targeting the NFATc1/Trem2 axis, whose function was significantly mitigated in both Trem2 and NFATc1 blocking experiments.

**Conclusion:**

Rhein promoted macrophage M2 polarization transition by targeting the NFATc1/Trem2 axis to regulate inflammation response and prognosis after ALI/ARDS, which shed more light on possibilities for the clinical treatments of this pathological process.

## Introduction

Acute lung injury (ALI) or acute respiratory distress syndrome (ARDS) is a severe acute respiratory failure, which occurs under many triggers including primary diseases and incentives [1]. It is well characterized by non-cardiogenic pulmonary edema and stubborn hypoxemia, with a high mortality rate [2, 3]. In recent years, the global pandemic of COVID-19 has led to the pathogenesis of ARDS obtaining a high level attention throughout the world [2, 4]. Even with all the effort that has been put in, the development of specific treatment plans and drug strategies for ALI/ARDS is still an important problem that so far urgently needs to be solved in the field.

Inflammation is accompanied by the whole range of pathological stages of ARDS, in which the relative abundance of pro- and anti-inflammatory cytokines determines the severity and prognosis of lung injury [5, 6]. As an essential part of the lung’s innate immune system, macrophages regulate the balance of pro- (e.g., interleukin 6 [IL-6], IL-1β, and tumor necrosis factor [TNF-a]) and anti-inflammatory cytokines (e.g., interleukins 4 [IL-4] and 10 [IL-10]) by changing the polarization state at different pathological stages [7]. In the swelling stage, M1 macrophages display the activation of NF-κB signaling and the high level of intracellular reactive oxygen species (ROS) to eliminate the focus while causing tissue destructions [8, 9],. In the proliferating stage, M1 and M2 macrophages undergo polar transformation, while in the regenerative stage, M2 macrophages mediate fibrosis repair [10–12]. Studies have indicated that the early transition of macrophage M2 polarization is beneficial to the prognosis of ALI/ARDS [13, 14]. Therefore, it may be an effective strategy to reduce the clinical severity and mortality of ALI/ARDS by means of intervention and the changing of the polarization state of macrophages at the early stage.

As a component of many traditional Chinese medicines (including rhubarb, aloe and senna leaves), rhein (4,5-dihydroxy-anthraquinone-2-carboxylic acid), also known as cassic acid, has been widely proved to display strong anti-inflammatory, anti-tumor, anti-fibrosis, antioxidant, and other pharmacological effects in many systems [15–18], as its treatment applications have been investigated in animal models involving chronic kidney disease [19, 20], intestinal inflammation [16, 21], liver cancer [22, 23], rheumatoid arthritis [24], Alzheimer’s disease [25], and osteoarthritis [26]. However, due to the identical pathological process of ALI/ARDS, it is unclear whether rhein could exhibit the same strong protective role. The purpose of this study was to investigate the effect of rhein on lipopolysaccharide (LPS)-induced ALI/ARDS, and to explore its in vivo and in vitro mechanisms as well as important targets involved in regulation.

## Methods and materials

### Animals and animal model

All animal experiments were ethically approved by Guizhou medical university. Mice were raised under SPF conditions and housed in temperature controlled on a 12 h light–dark cycle. *Trem2*^−/−^ (C57BL/6 J-*Trem2*^*em2Adiuj/J*^) mice with C57BL/6 J background (027197) were purchased from Jackson Laboratory, USA. For ALI/ARDS modeling, in accordance with the experimental design, the C57BL/6 J mice or *Trem2*^−/−^ mice (8-week-old, male) were intratracheally instilled with LPS (3 mg/kg, 50 μL per animal), while animals in the control group were treated with the same volume of solvent (normal saline) [27]. In terms of rhein treatment, the final volumes of rhein with 50 mg/kg and 100 mg/kg were intraperitoneal applied for 2 consecutive days (the modeling time and 24 h after modeling) and control group received equal amount of vehicle (40% PEG 400-PBS solution by sonication) [28]. For the application of NFATc1 blocker (NFATc1-IN-1), 10 mg/kg NFATc1-IN-1 was administered the same way as rhein did. Mice were sacrificed for further analysis 48 h after modeling. In each group, six animals were used for the collection of the bronchoalveolar lavage fluid (BALF). For another six mice, their lungs were used for the analysis of Evans blue albumin. For the other six mice, their left lungs were used for the measurements of wet-to-dry ratio, with their right lung fixed with 4% paraformaldehyde for the following histological analysis [29].

### Reagents and antibodies

The LPS (strain O111:B4) was purchased from Sigma (Sigma, L2630, USA). The rhein of analytical standard with its concentration ≥ 98% was purchased from Macklin (Macklin, R817293, China). The NFATc1 inhibitor NFATc1-IN-1 was purchased from MedChemExpress (MCE, HY-147369, USA). Biotinylated-Rhein (Bio-Rhein) and biotin were purchased from Bocong Biotech (Guangzhou, China). The anti-P65 (CST, 8242, USA), anti-phospho-P65(CST, 3033, USA), anti-CD68 (abcam, ab283654, USA), anti-CD86 (abcam, ab220188, USA), anti-Mannose Receptor (CD206) (abcam, ab64693, USA), anti-Trem2 (abcam, ab86491, USA), anti-EpCAM (Abclonal, A19301, China) anti-β-actin (CST, 3700, USA), and anti-histone H3 (CST, 4499, USA) antibodies were used for western immunoblotting or immunofluorescence staining in accordance with the experimental requirements.

### Survival curve

For the survival experiments, ten mice was used for every treatment group with their survival status recorded every 12 h. After 7 days, animals that were still alive were anesthetized with 2% sodium pentobarbital and sacrificed by cervical dislocation.

### Histology and immunostaining

The mouse right lung tissues underwent sequential 24 h of 4% paraformaldehyde fixation, paraffin embedding, and sectioning into 6-μm pieces. Then the sections were stained with hematoxylin and eosin (H & E, Servicebio, China). Those for immunofluorescence staining, were permeabilized with 0.1% Triton X-100 and blocked with 10% horse serum (Gibco) [30]. Primary antibodies were stained at 4 °C overnight, AF488- and AF594-labeled secondary antibodies were used for labeling the primary antibodies (1:1000, Invitrogen) in immunofluorescence staining. DAPI (2 μg/ml, Beyotime, China) was used for the nuclei labeling for 3 min.

### Enzyme-linked immunosorbent assay (ELISA)

ELISA was carried out to measure the concentrations of TNF-α and IL-1β in BALF. The experiments were carried out in accordance with the manufacturers’ instructions. Mouse TNF-α (Beyotime, PT512, China), Mouse IL-1β (Beyotime, PI301, China), Mouse IL-4 (Beyotime, PI612, China) and Mouse IL-10 (Beyotime, PI522, China) ELISA kits were used for this study.

### Lung wet-to-dry ratio

The lung wet-to-dry (W/D) ratio was conducted to evaluate the extent of lung edema and permeability of alveolar-capillary membrane. The wet weight was measured immediately after the excision of the lung, while after drying at 60 °C for 48 h, the dry weight was recorded. The lung W/D ratio was calculated as wet weight/dry weight.

### Evans blue albumin

An Evans blue dye-labeled albumin mixture (EB, 40 mg/kg, Sigma) was injected into the left jugular vein of each mouse 45 min before the mice were sacrificed. Then steps were followed as previously reported. Extravasated EB concentration in the lung homogenate was presented as micrograms of Evans blue dye per gram of tissue.

### TUNEL assay

By following the instructions (Beyotime, C1086, China), lung sections were incubated with terminal deoxynucleotide transferase-mediated dUTP nick end labeling (TUNEL) reagent containing terminal deoxynucleotidyl transferase and fluorescent isothiocyanate dUTP, followed by antigen retrieval.

### Lung index and lung damage score

By following the previous study [31], the lung index was shown in the following formula: lung index = lung weight (g)/body weight (g) × 100%. The pathological changes of each lung section (lung damage score) were represented as the mean of ten different fields at the level of 0–3 (0: normal, 1: mild, 2: moderate, 3: severe).

### Biotin pull-down assay

For biotin pull-down assays, the Pierce™ Biotinylated Protein Interaction Pull-Down Kit was used (Thermo Fisher, 21,115, USA). 100 μL of 10 mM biotinylated-Rhein was added to 50 μl streptavidin beads and incubated at 4 °C for 1 h. For control group, biotin alone was used. Lysates from RAW264.7 cells were used for the following pull-down experiment.

### Chromatin immunoprecipitation (ChIP)-quantitative PCR

ChIP assays were performed using RAW264.7 cells transfected with an Nfatc1 expressing or mock vector with a ChIP-IT express kit (Active Motif), in accordance with the manufacturer's instructions. The cells were cross-linked with 1% formaldehyde for 15 min and neutralized via glycine with the terminal concentration of 0.125 M. The cell pellets were sonicated and immunoprecipitated with administration of magnet beads coated by V5 antibody (M15-11, MBL) for 6 h at 4 °C. Cross-linking was reversed using a reverse cross-linking buffer, and then DNA fragments were analyzed by qRT-PCR using the following primer pairs: Trem2 pro, 5′-AAGAGCCAGGTAATGCACCT-3′ and.

5′-GGTCCAGGCACAGGGTC-3′;

### Western immunoblotting

Protein samples of lung tissue, RAW264.7 cell line, and biotin-pull down were collected and isolated with RIPA buffer (Beyotime, P0013B, China), with the treatment conditions as designed in accordance with the experiments. The nuclear and cytoplasmic extraction kit (Thermo, 78,833, USA) was used for the separation of nuclear and cytoplasmic proteins. The protocol was followed as described previously [32].

### Quantitative real-time PCR (qRT-PCR)

Total RNAs were isolated with RNAiso Plus reagent (TaKaRa, 9109, Japan) from RAW264.7 cells according to the instructions. Further steps followed the previous research [33]. The primer sequences were as follows: mouse β-actin, 5′-GGCTGTATTCCCCTCCATCG-3′ and

5′-CCAGTTGGTAACAATGCCATGT-3′;mouse Tnf, 5′-CCCTCACACTCAGATCATCTTCT-3′and

5′-GCTACGACGTGGGCTACAG-3′;mouse Il1b, 5′- GCAACTGTTCCTGAACTCAACT-3′ and

5′- ATCTTTTGGGGTCCGTCAACT-3′;mouse iNOS, 5′- GTTCTCAGCCCAACAATACAAGA-3′and

5′- GTGGACGGGTCGATGTCAC-3′;mouse CD86, 5′- TGTTTCCGTGGAGACGCAAG-3′and

5′- TTGAGCCTTTGTAAATGGGCA-3′.

### Trem2 knockdown in RAW264.7 cells

For Trem2 knockdown, the pLV-shTrem2 lentivirus expressing vector was applied for the packaging of lentivirus, with the oligo inserted into the MCS site of the vector (5′-GGAATCAAGAGACCTCCTTCCTTCAAGAGAGGAAGGAGGTCTCTTGATTCC-3′). Using the three plasmids system (pol/gag, VSV-G and pLV-Puro/pLV-shTrem2), lentivirus was packaged in 293 T cell line and underwent purification before transfection. Then LV-Puro and LV-shTrem2 were applied for the infection of RAW264.7 cell line with the MOI set at 100. 48 h after infection, cells were screened with puromycin and termed as LV-Puro or LV-shTrem2 with the former group set as control.

### ALI/ARDS microenvironment-simulated macrophage polarization

The mouse alveolar epithelial cell line MLE-12 was stimulated with *E.coli*-LPS (100 ng/ml) for 48 h at 37 °C in a 5% CO_2_ atmosphere. Then the supernatant was collected and used as the culture medium for RAW264.7 macrophage cell line with the duration of 48 h.

### Measurement of intracellular ROS level

The Dihydroethidium (DHE) Cellular ROS Detection Assay Kit (BB-47051–1, Bestbio, China) was used to measure the ROS level in macrophages in accordance with the manufacturers’ instructions. The fluorescence intensity was measured using 100 μL cell lysates (0.5% Triton X-100) at stimulation time point of 30 min on a hybrid multi-mode microplate reader (Synergy H1, BioTek) using the excitation/emission wavelengths of 535/610 nm.

### Luciferase reporter assay

In accordance with the experimental designs, RAW264.7 cell line was co-transfected with NFATc1 expressing vector, Trem2-wt or Trem2-mut responsive reporter plasmids (pGL-Trem2-wt-Luc or pGL-Trem2-mut-luc) to validate the transcription regulating role of NFATc1 on the promoter region of Trem2. In another part, RAW264.7 cell line was transfected transiently first with si-NC and si-Nfact1. And these two groups would be co-transfected transiently with Trem2-responsive reporter plasmid (pGL-Trem2-Luc) with Renilla luciferase expression plasmid (phRL-TK) using Lipofectamine 48 h after the first transfection. After 24-h post-transfection, the cells were harvested following different experiments and stimulation for the measurement of luciferase activity using the Dual-Glo Luciferase Assay System (Promega, Madison, WI, USA). Specific Trem2 luciferase activity was normalized to that of the internal control.

### RNA sequencing

Total RNA was isolated from the RAW264.7 cells cultured by conditioned medium, with the treatment of 25 μM of rhein or vehicle using TRIzol reagent (Invitrogen) according to the manufacturers’ instructions. The measurement of RNA integrity, the library preparation, and RNA sequencing process were carried out as previously reported [34]. The heatmap displayed the top 100 upregulated genes (TPM > 10, padj < 0.05, Log2FC > 1.5). The volcano plot depicted all genes with TPM > 0. And all genes with TPM > 0 were taken into the calculation of PCA and gene set enrichment analysis using the database of KEGG pathway (c2.cp.kegg. v6.0.symbols.gmt).

### SuperPred target prediction

SuperPred was applied for large-scale targets screening, which is a prediction webserver for ATC code and target predicition of compounds [35]. Predicting ATC codes or targets of small molecules and, thus, gaining information about the compounds offers assistance in the drug development process. The webserver's ATC predicition as well as target prediction is based on a machine learning model, using logistic regression and Morgan fingerprints of length 2048.

### Molecular docking

The crystal structures of NFATC1 protein used for docking were obtained from the UniProt database (UniProt ID: O88942 and the included AlphaFold2 was used to predict the conformation). The structure of small molecule rhein was obtained from PubChem database (PubChem ID: 10,168). Before docking, AVOGADR 1.2.0 is used to minimize the energy of the 3D structure of small molecule rhein under MMFF94 force field. In this study, AutoDock Vina 1.1.2 was used for molecular docking [36]. Before docking, the academic open source version of PyMol was used to hydrotreate the target protein [37]. Then the ADFRsuite 1.0 was applied for converting the molecule and target protein into the PDBQT format necessary for AutoDock Vina 1.1.2 docking. Before docking, take the centroid of the protein as the center of the box (see Table 1 for the central coordinates of the protein), adjust the appropriate X, Y, Z side lengths to build the box, and make it fully wrap the entire protein (see Table 1 for the X, Y, Z side length parameters of the docking box). During docking, the grid box and PDBQT files of processed proteins and small molecules were used as input files, and Vina was used for docking. The global search details of docking were set to 32, with the other parameters remained default settings. Finally, the docking conformation with the highest score output was considered as a combination conformation, and the academic open source version of PyMol was used to visually analyze the docking results. Ligplot 2.2.4 was applied to analyze the combination mode (interaction force) between the small molecule and target protein.

**Table 1:** The central coordinates of protein and docking box parameters

Protein	Center coordinates(X, Y, Z)	Side length(X, Y, Z)
NFATC1	− 10.905, 3.044, − 7.455	126.0, 126.0, 126.0

### Data and statistical analysis

The results were all presented as the means ± SDs. A normal distribution test was performed to determine whether a parametric or non-parametric test was conducted. Student’s t test was used for comparisons between two groups. Comparisons of more than three groups were carried out by one-way ANOVA. All statistical analyses were performed using GraphPad Prism 8.0 software. Differences were regarded as significant when *p* < 0.05.

## Results

### Rhein alleviates LPS-induced ARDS in vivo

**Fig. 1:**
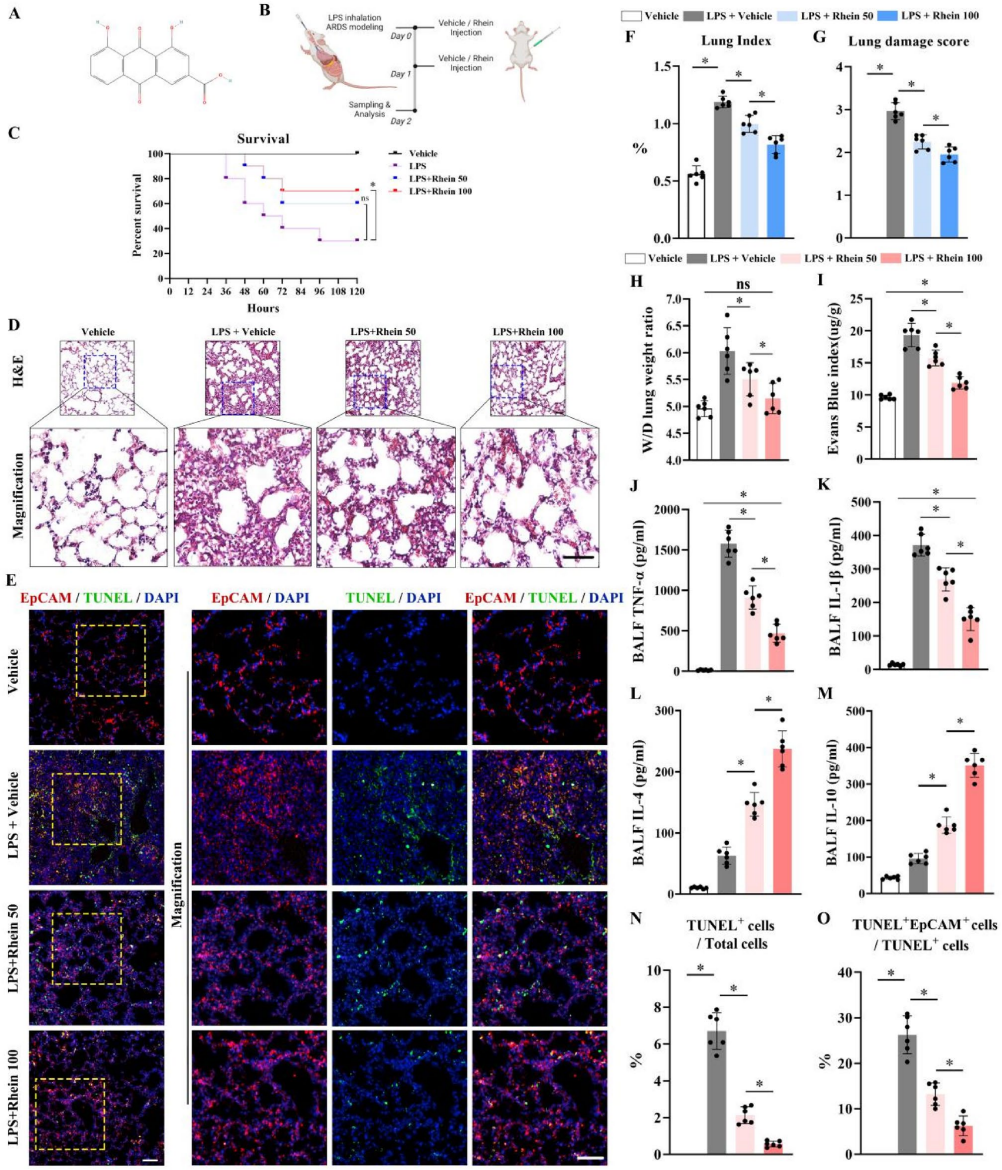
Rhein alleviated LPS-induced lung inflammation and injury in mice. **A** The chemical structure of rhein. **B** Schematic diagram of ALI/ARDS modeling and rhein administration. **C** Kaplan–Meier survival curve of ALI/ARDS with different treatments (*n* = 10 per group). **D** Representative images of H&E stained lung tissues and magnified views (upper scale bar = 100 μm, lower scale bar = 50 μm; blue dotted box indicating the zoomed area; *n* = 6 per group). **E** Representative images of immunofluorescence staining by labeling epithelial cells (Epcam, red), apoptotic cells (TUNEL, green) and nuclei (DAPI, blue) (left scale bar = 100 μm; right scale bar = 50 μm; yellow dotted box indicating the zoomed area; *n* = 6 per group). **F** Evaluated lung index (*n* = 6 per group). **G** Evaluated lung damage score (*n* = 6 per group). **H** Wet-to-dry (W/D) lung weight ratio (*n* = 6 per group). **I** Evans blue index (*n* = 6 per group). **J–M** Concentrations of TNF-α, IL-1β, IL-4, and IL-10 in the bronchoalveolar lavage fluid (BALF) (*n* = 6 per group). **N** Statistics of the proportion of TUNEL-positive apoptotic cells versus total cells (*n* = 6 per group). **O** Statistics of the proportion of TUNEL-positive epithelial cells (TUNEL^+^EpCAM^+^) versus total apoptotic cells (*n* = 6 per group). ns, no significance; *P < 0.05. means ± SD (Color figure online)

The chemical structure of rhein is shown in Fig. 1A. To investigate its role in acute respiratory distress syndrome (ARDS), the animal model of ARDS was established by LPS inhalation at Day 0, followed by vehicle or rhein intraperitoneal injection for 2 consecutive days (including Day 0), before all animals were sacrificed on Day 2 for further analysis (Fig. 1B). For the discovery of whether long-term protective roles of rhein would exist in treating ALI/ARDS, the survival study was carried out. Results indicated that rhein administration with the dose of 100 mg/kg would significantly upregulate the long-term survival after modeling (Fig. 1C). Hematoxylin and eosin (HE) staining showed that LPS administration resulted in severe intra-alveolar and interstitial edema and infiltration of inflammatory cells, including a decreased number of alveoli. By contrast, rhein alleviated the inflammatory responses and alveolar damages in a dose-dependent manner (Fig. 1D). Immunofluorescence staining (IF) showed that the application of rhein significantly reduced apoptotic epithelial cells (EpCAM^+^TUNEL^+^ cells), indicating the inhibitory effect of rhein on the inflammation infiltration and the damage of lung stromal cells (Fig. 1E, N, O)). In addition, the downregulated lung index with the administration of rhein indicated the reduced permeability of capillaries when compared with vehicle administered only (Fig. 1F). The downregulated lung damage score (Fig. 1G), the wet/dry lung weight ratio (Fig. 1H), and Evans blue index (Fig. 1I) also proved the protective roles of rhein in alleviating the intra-alveolar and interstitial edema. Besides, the molecules concerning the M1 (TNF-α, IL-1β) and M2 (IL-4, IL-10) polarization of macrophages were measured in BALF. It was observed that the concentrations of TNF-α and IL-1β were downregulated, along with the upregulated IL-4 and IL-10 levels, indicating the alleviated inflammation and the advance of regenerative stage (Fig. 1J–M).

### Rhein suppressed the LPS-induced M1 polarization and ROS production in ALI/ARDS

**Fig. 2:**
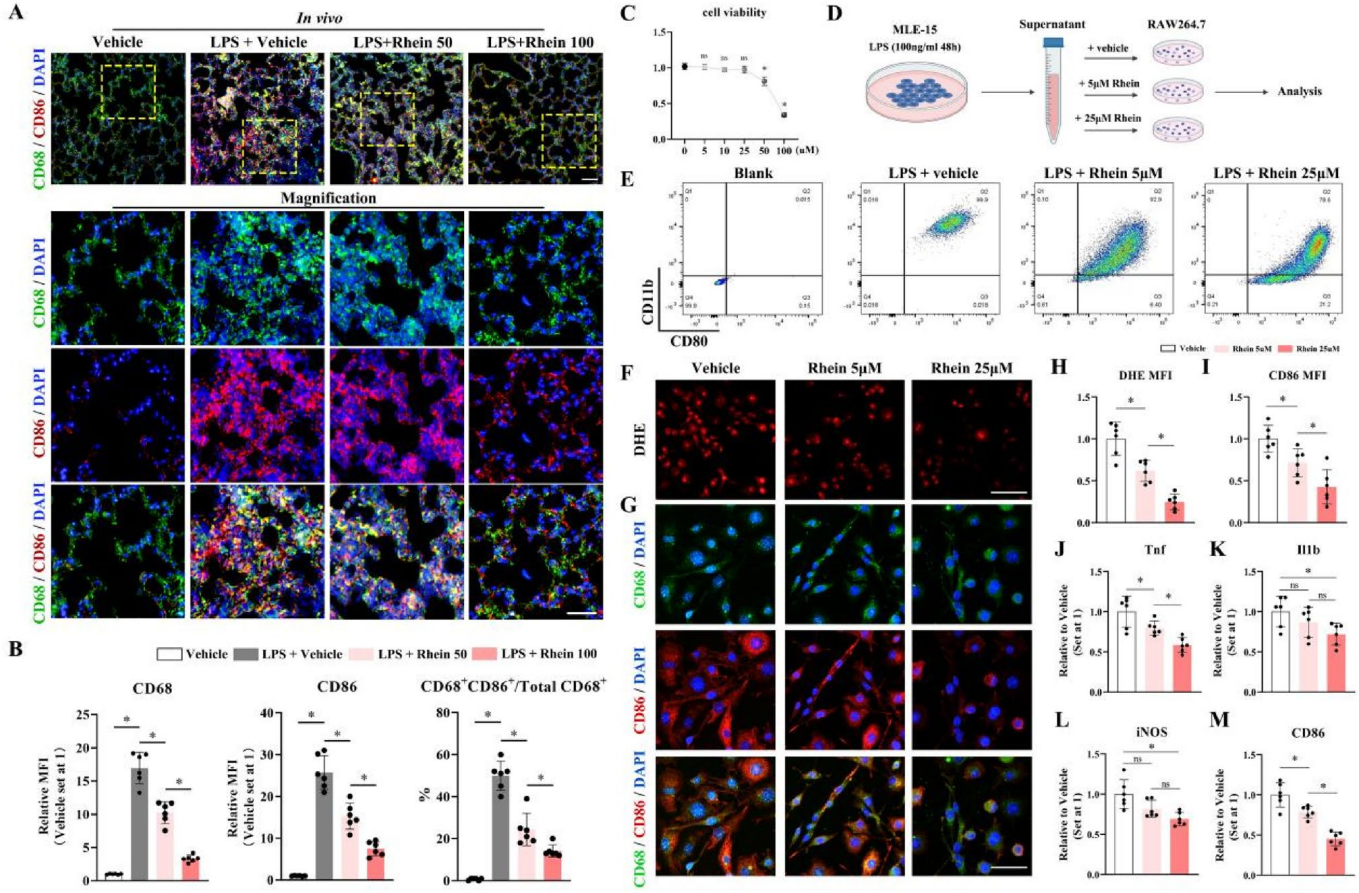
Rhein suppressed M1 polarization of macrophages in ALI/ARDS modeling in vivo and in vitro. **A** Representative images of immunofluorescence staining by labeling total macrophages (CD68, Green), M1 macrophage marker (CD86, red) and nuclei (DAPI, blue) (upper scale bar = 100 μm; lower scale bar = 50 μm; yellow dotted box indicating the zoomed area; *n* = 6 per group). **B** Statistics of mean fluorescence intensity (MFI) of CD68 and CD86, along with the proportion of M1 macrophages, indicated by CD68^+^CD86^+^ cells versus total CD68^+^ cells (*n* = 6 per group). **C** Cell viability assay with different rhein concentrations (*n* = 5 per concentration). **D** Schematic diagram of mimicking ALI/ARDS microenvironment in vitro. **E** Representative images of flowcytometry by labeling M1 macrophages (CD11b^+^CD80^+^)in vitro with different treatments (*n* = 3 per group). **F** Representative images of DHE assay to measure the intracellular ROS level (scale bar = 50 μm; *n* = 6 per group). **G** Representative images of immunofluorescence staining by labeling with CD68 (green), CD86 (red), and nuclei (DAPI, blue) (scale bar = 25 μm; *n* = 6 per group). **H, I** Statistics of mean fluorescent intensity (MFI) of DHE assay and CD86 signal. **J**–**M** RT-qPCR quantification of Tnf, IL-1β, iNOS, and CD86 mRNA expression levels (*n* = 6 per group). *P < 0.05. means ± SD (Color figure online)

To investigate whether rhein affects the polarization of macrophages, which were abundant in infiltrating inflammatory cells, in ALI/ARDS, immunofluorescence staining and flow cytometry were conducted. In vivo, the administration of rhein significantly attenuated the M1 polarization of macrophages in a dose-dependent manner (Fig. 2A, B). In vitro, the toxic effect of rhein on the viability of the RAW264.7 cell line was tested by CCK-8. Rhein would not yield a significant toxic effect on the cell viability until the concentration reached 50 μM (Fig. 2C). Thus, 5 μM and 25 μM were chosen as representative concentrations for further in vitro study. To simulate the in vivo pathological process of ALI/ARDS, we conducted the in vitro study by first treating the pulmonary epithelial cell line (MLE-15) with LPS (100 ng/ml) for 48 h. Then the conditioned media of LPS-stimulated MLE-15 cells were collected and the RAW264.7 cell line was treated by this media with further additions of vehicle or rhein (5 μM and 25 μM) (Fig. 2D). Given the evidence that the intracellular signal of oxidative species played a pivotal role in regulating macrophage polarization, the DHE assay reflected the total ROS level was carried out in vitro. It demonstrated that rhein significantly reduced the LPS-induced high level of total ROS (Fig. 2F, H), along with the alleviated M1 polarization, indicated by flow cytometry (percentage of CD11b^+^CD80^+^ cells) and immunofluorescence staining (Fig. 2G, I). Further, the inflammation-related genes, Tnf (encoding TNF-α), Il1b (encoding IL-1β), and iNOS expression followed a similar pattern (Fig. 2J–M). Collectively, these data suggested that rhein mitigated the M1 polarization of macrophages in LPS-induced ALI/ARDS.

### Trem2 was significantly enriched in rhein-induced inhibitory effects on LPS-induced ALI/ARDS by RNA sequencing

**Fig. 3:**
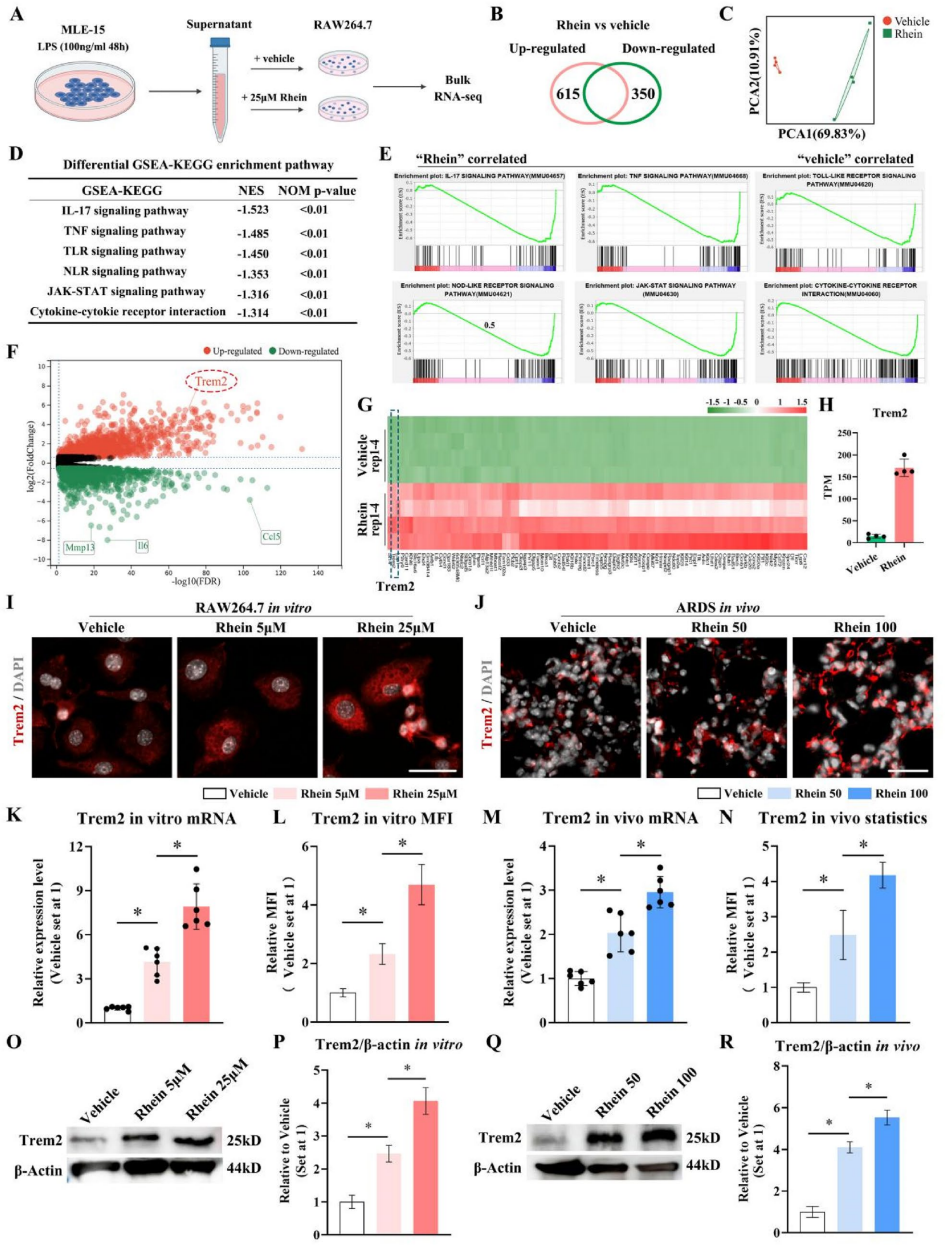
Trem2 was significantly upregulated in rhein-treated group.** A** Schematic diagram of grouping strategy for bulk RNA sequencing. **B** Venn diagrams of the number of differentially expressed genes. **C** PCA of the samples. **D** GSEA using the KEGG database. **E** Enrichment plot of representative pathways. **F** Volcano plots of the differentially expressed genes. **G** Heat map of the top 100 upregulated genes in the rhein treated group as compared to vehicle (*n* = 4 per group). **H** TPM analysis of Trem2 with the treatment of vehicle or rhein, defined by RNA-seq (*n* = 4 per group). **I** Representative images of immunofluorescence staining of RAW264.7 by labeling Trem2 (red) and nuclei (DAPI, grey) (scale bar = 25 μm; *n* = 6 per group). **J** Representative images of immunofluorescence staining of lung tissues with ALI/ARDS modeling by labeling Trem2 (red) and nuclei (DAPI, grey) (scale bar = 50 μm; *n* = 6 per group). **K, M** RT-qPCR quantification of Trem2 mRNA expression levels in ARDS in vivo and in vitro (*n* = 6 per group). **L**–**N** Statistics of mean fluorescent intensity (MFI) of Trem2 signal (n = 6 per group). **O–P** Protein level of Trem2 in vitro measured by WB (*n* = 3 per group). **Q-R** Protein level of Trem2 in vivo measured by WB (*n* = 3 per group). *P < 0.05. means ± SD (Color figure online)

To identify the variations in transcription level by which rhein attenuated inflammation, bulk RNA sequencing (RNA-seq) of RAW264.7 cells cultivated under inflammation-induced conditioned media with or without rhein was conducted (Fig. 3A). For data analysis, |Log_2_FC|≥ 1.5 and *p*.adj ≤ 0.05 were considered statistically significant. A total of 965 genes, including 615 upregulated and 350 downregulated genes, exhibited different expression patterns with or without rhein (Fig. 3B). The principal component analysis (PCA) showed remarkable disparity occurred with the applications of rhein (Fig. 3C). The gene set enrichment analysis (GSEA) indicated that the downregulated pathways were enriched in IL-17, TNF, TLR, NLR, JAK-STAT signalings and cytokine–cytokine receptor interactions in accordance with the KEGG database (Fig. 3D, E). The volcano map illustrated the differential expressed genes, in which the top 100 upregulated were listed in the heatmap, indicating Trem2 to be a significant upregulated gene in the rhein-treated RAW264.7 cells (Fig. 3F, G, H). To validate the result of RNA sequencing, the immunofluorescence staining, RT-qPCR, and western blot were carried out to measure the expressing pattern of Trem2 in ARDS, with the administration of vehicle or rhein of different concentrations. It was observed that the application of rhein would yield significant upregulation of Trem2 in both mRNA and protein levels in a dose-dependent manner, both in vitro (Fig. 3, I, K, L, O, P) and in vivo (Fig. 3J, M, N, Q, R). These results suggested that the protective roles of rhein in the pathological process of ALI/ARDS might be related with the elevated Trem2 level.

### Trem2 knockdown mitigated the rhein-mediated anti-inflammatory role in the ARDS model in vitro

**Fig. 4:**
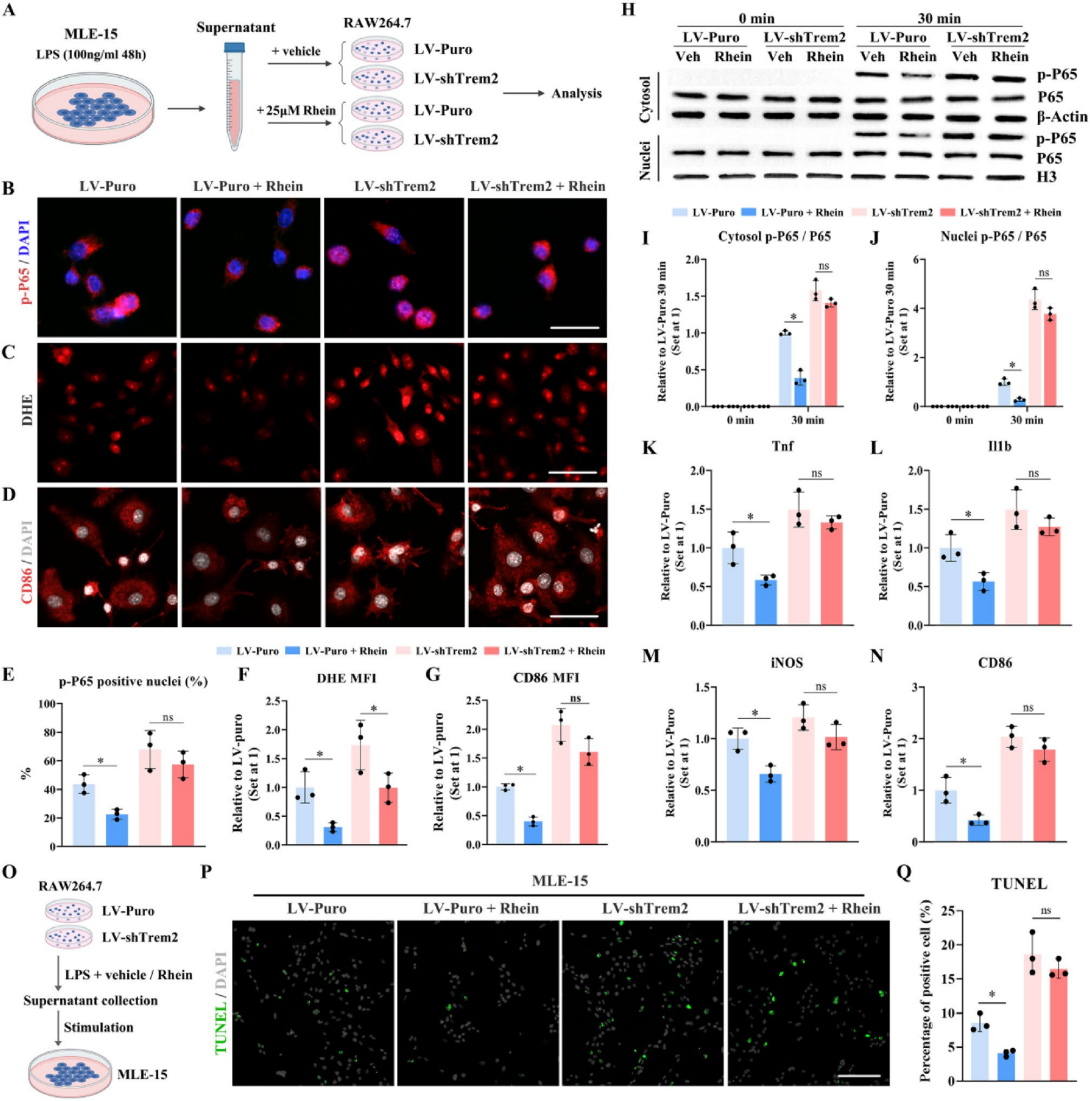
Trem2 deficiency mitigated the rhein-mediated anti-inflammatory effects in vitro. **A** Schematic diagram of experimental design. **B** Representative images of immunofluorescence staining by labeling p-P65 (red) and nuclei (DAPI, blue) (scale bar = 25 μm; *n* = 6 per group). **C** Representative images of DHE assay to measure the intracellular ROS level (scale bar = 50 μm; *n* = 6 per group). **D** Representative images of immunofluorescence staining by labeling CD86 (red) and nuclei (DAPI, grey) (scale bar = 25 μm; *n* = 6 per group). **E** Quantification of p-P65-positive nuclei. **F** Quantification of MFI of **C**.** G** Quantification of MFI of **D**. **H–J** Expression and phosphorylation of P65 in cytosol and nuclei measured by WB (*n* = 3 per group). **K–N** RT-qPCR quantifcation of Tnf, IL-1β, iNOS, and CD86 mRNA expression levels (*n* = 6, per group). **O** Schematic diagram of the strategy for stimulating MLE-15. **P** Representative images of TUNEL assay to measure the apoptotic MLE-15 cell with different treatment (*n* = 3 per group). **Q** Quantification of the percentage of TUNEL-positive cells. *P < 0.05. means ± SD (Color figure online)

To determine whether rhein played its inhibitory role through Trem2, the shTrem2-based mRNA knockdown and evaluation of the downstream NF-κB signaling were carried out (Fig. 4A). P65, as the key molecule of NF-κB pathway, could react to various pathological stimuli and is involved in many inflammatory and even cancer processes. Via western immunoblotting and immunofluorescence, both nuclei or cytosol p-P65 significantly declined 30 min after the applications of conditioned media in the LV-Puro group (control group) with rhein pretreatment. However, in stark contrast, the inhibition of Trem2 led to no significant changes of p-P65 in nuclear and cytoplasmal level after rhein was administered when compared with the vehicle, indicating that Trem2 silencing significantly neutralized the inflammation inhibitory effect of rhein (Fig. 4B, H, I, J). Moreover, pro-inflammatory and M1 macrophage-related genes, including Tnf, Il1b, iNOS, and CD86 also underwent a similar change after Trem2 knockdown (Fig. 4K–N). In addition, DHE assay and immunofluorescent staining also showed that both the intracellular ROS level and CD86 signal in the shTrem2 group remained nearly unchanged even with rhein pretreatment. By contrast, both parameters plummeted to a relatively low level in the control group which was treated with rhein (Fig. 4C–G). In addition, the supernatants of LV-puro and LV-shTrem2 stably infected RAW264.7 cell lines, with the stimulation of LPS, rhein (25 μM) or vector were applied for co-culturing with MLE-15 cell line (Fig. 4O). It was discovered that the proportion of apoptotic MLE-15 cells declined in the LV-puro group with rhein treatment. While, in the Trem2 knockdown group, no significant changes in the apoptotic cell number were observed whether rhein was administered or not, suggesting that rhein played its inflammatory regulatory roles in a Trem2-dependent manner.

### Trem2 knockout attenuated the rhein-mediated anti-inflammation effects on ARDS model in vivo

**Fig. 5:**
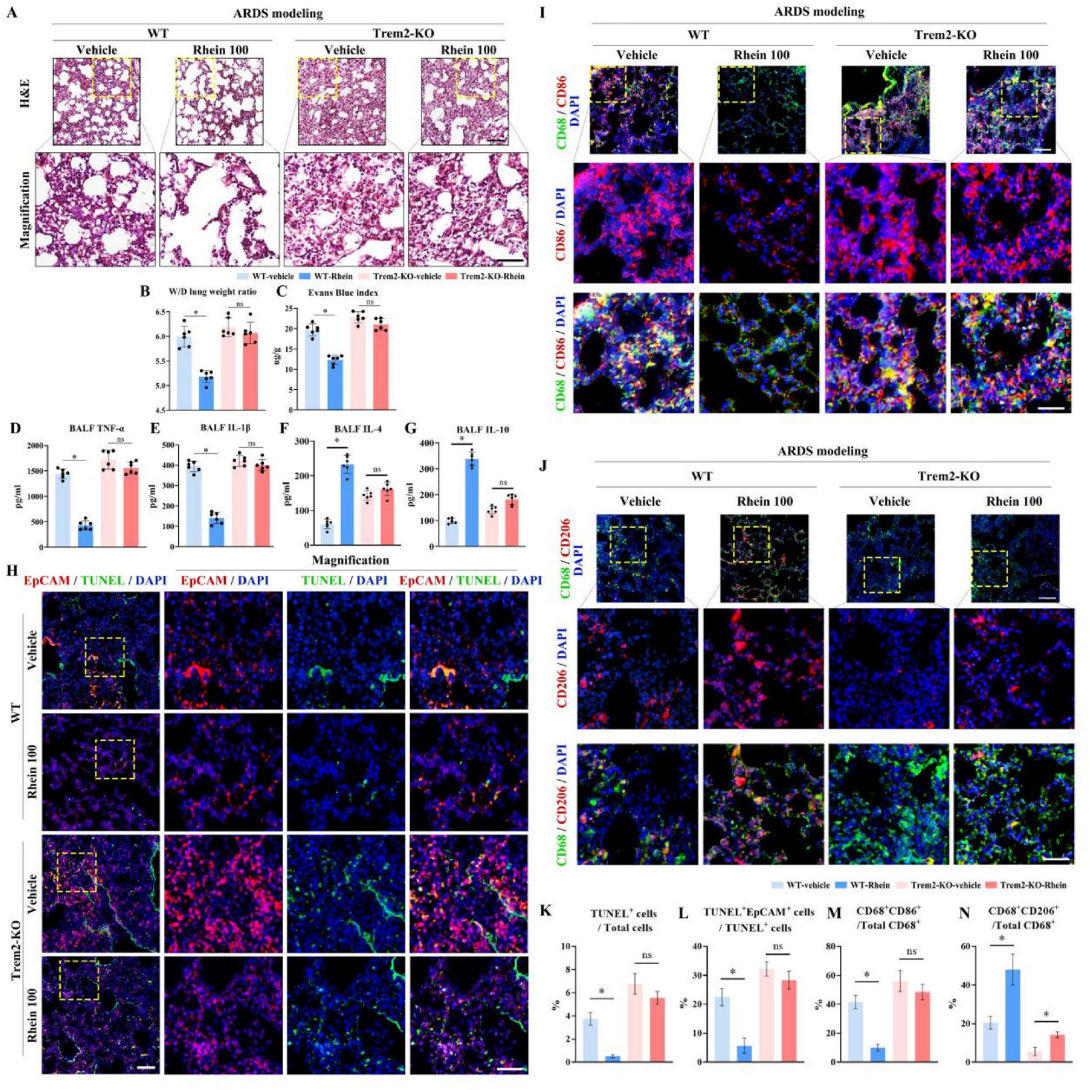
Trem2 knockout attenuated the rhein-mediated anti-inflammatory effects in ALI/ARDS in vivo. **A** Representative images of H&E stained lung tissues with zoomed views below (upper scale bar = 100 μm; lower scale bar = 50 μm; yellow dotted box indicating the zoomed area; *n* = 6 per group). **B** Wet-to-dry (W/D) lung weight ratio (*n* = 6 per group). **C** Evans blue index (*n* = 6 per group). **D–G** Concentrations of TNF-α, IL-1β, IL-4, and IL-10 in the bronchoalveolar lavage fluid (BALF) (*n* = 6 per group). **H** Representative images of immunofluorescence staining by labeling epithelial cells (Epcam, red), apoptotic cells (TUNEL, green), and nuclei (DAPI, blue) (left scale bar = 100 μm; right scale bar = 50 μm; yellow dotted box indicating the zoomed area; *n* = 6 per group). **I** Representative images of immunofluorescence staining labeling M1 macrophages via CD68 (green), CD86 (red), and nuclei (DAPI, blue) (upper scale bar = 100 μm; lower scale bar = 50 μm; *n* = 6 per group). **J** Representative images of immunofluorescence staining labeling M2 macrophages with CD68 (green), CD206 (red), and nuclei (DAPI, blue) (upper scale bar = 100 μm; lower scale bar = 50 μm; *n* = 6 per group). **K, L** Statistics of the proportion of TUNEL-positive apoptotic cells versus total cells and TUNEL-positive epithelial cells (TUNEL^+^EpCAM^+^) versus total apoptotic cells (*n* = 6 per group). **M, N** Statistics of the proportion of M1 and M2 macrophages by calculating CD68^+^CD86^+^ or CD68^+^CD206^+^ cells versus total CD68^+^ cells (*n* = 6 per group). *P < 0.05. means ± SD (Color figure online)

To clarify whether rhein played its regulatory role via Trem2 in vivo, the LPS-induced ARDS model was established on wild-type and Trem2-knockout (Trem2^−/−^) mice respectively. In WT mice, the inflammatory pathological changes were significantly ameliorated by rhein administration. In contrast, the knockout of Trem2 in mice greatly attenuated the anti-inflammation effects of rhein on ARDS model (Fig. 5A). The parameters of lung inflammation and pro-inflammatory cytokines, including wet/dry lung weight ratio, Evans blue index, and TNF-α, IL-1β concentrations in BALF changed in a similar pattern (Fig. 5B–E). For the M2 polarization-related molecules, the reduced levels of IL-4 and IL-10 indicated the anti-inflammatory role of rhein was mitigated when Trem2 lost its function (Fig. 5F–G). Via IF staining, it was observed that rhein significantly reduced the apoptotic epithelial cells (EpCAM^+^TUNEL^+^ cells) in WT mice, while this effect was blunted in the Trem2-KO group (Fig. 5H, K, L). In addition, rhein reduced the CD68^+^CD86^+^ cells (M1 macrophages) (Fig. 5I, M) and promoted the macrophage M2 polarization transition (CD68^+^CD206^+^) (Fig. 5J, N) in the inflammatory lung areas. By contrast, the anti-inflammation effect of rhein in ARDS was offset with Trem2 knockout in vivo, indicating a Trem2-dependent manner.

### Rhein: its anti-inflammatory effects via interacting with NFATc1, the upstream transcription factor of Trem2

**Fig. 6:**
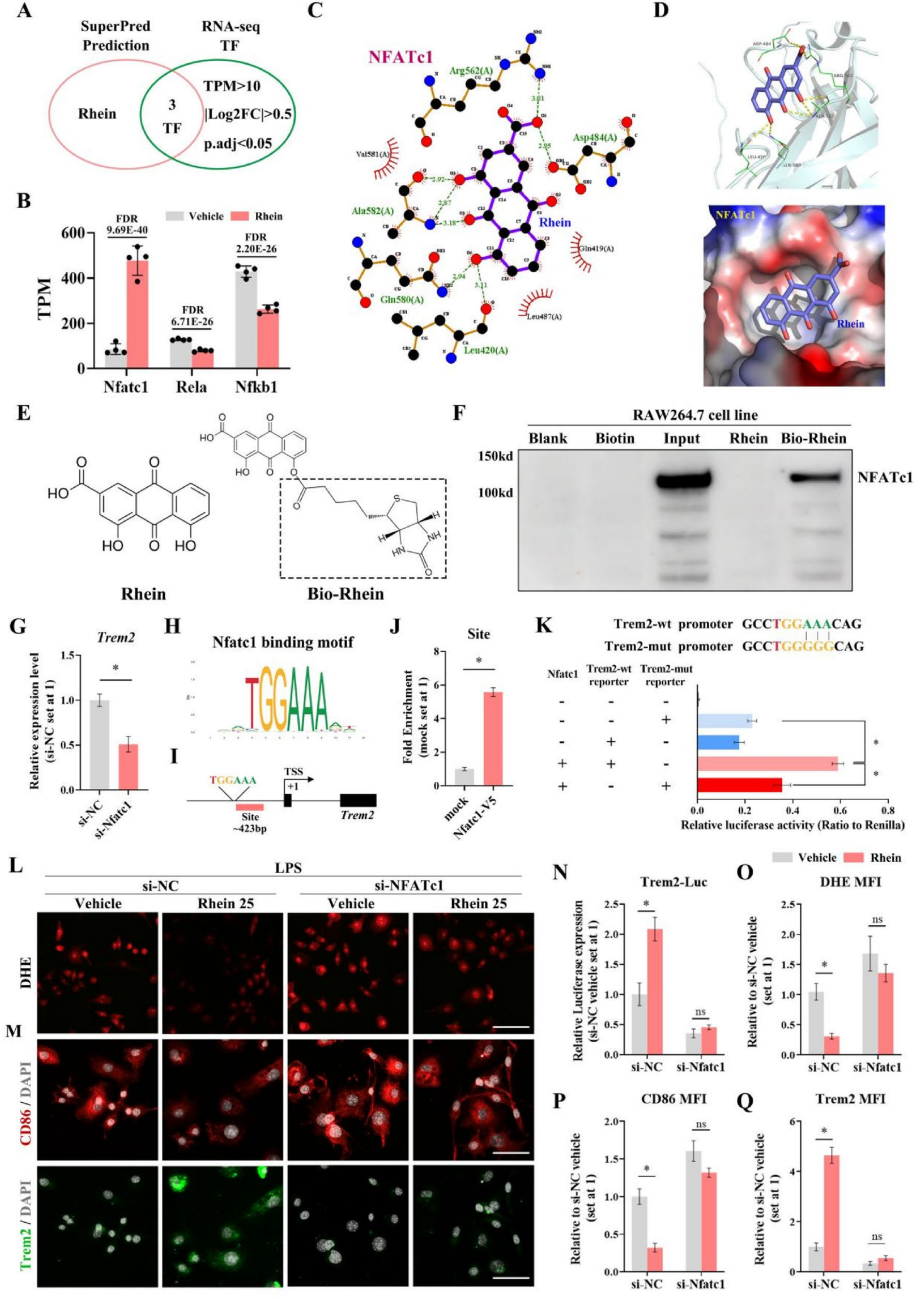
Rhein plays its anti-inflammatory role by interacting with NFATc1, the upstream transcription factor of Trem2.** A** Screening strategy of target transcription factor (TF). **B** TPM of screened TFs. **C** 2D interaction diagram of NFATc1-rhein. **D** Localized views of NFATc1-rhein composites obtained by molecular docking. **E** Chemical structure of rhein and biotin-labeled rhein (Bio-Rhein). **F** Biotin pull-down assay using RAW264.7 lysates. **G** qRT-PCR analysis of Trem2 expression in RAW264.7 cells transfected with si-NC or si-NFATc1 (*n* = 3 per group). **F** Mouse NFATc1 binding motif (MA0624.2 from JASPAR database). **G** Diagram of binding sites of NFATc1 on Trem2 promoter region. Genomic PCR was performed using primer sets for site (− 521 bp to − 360 bp) in the Trem2 promoter. **J** ChIP analysis of NFATc1 enrichment of Trem2 promoter in RAW264.7 cells transfected with mock or NFATc1-V5. **K** Sequences of wild-type Trem2 promoter and mutant promoter region. Trem2 promoter reporter activities were evaluated in RAW264.7 cells transfected with NFATc1 expression vector. **L** Representative images of DHE assay to measure the intracellular ROS level (scale bar = 50 μm; *n* = 6 per group). **M** Representative images of immunofluorescence staining by labeling CD86 (red), Trem2 (green), and nuclei (DAPI, grey) (scale bar = 25 μm; *n* = 6 per group). **N** Transcription activity of Trem2 measured by Trem2 luciferase (*n* = 3 per group). **O–Q** Quantifications of MFI of **L** and **M**. *P < 0.05. means ± SD (Color figure online)

To screen out the target transcription factor (TF) protein of rhein in this model, the combination use and analysis of bulk RNA-seq and SuperPred was carried out with identical parameters, including TPM > 10, |Log2FC|> 0.5 and *p*.adj < 0.05 (Fig. 6A). A total of three TF genes were screened out, including NFATc1, Rela, and Nfkb1. Among the three genes encoding TFs, NFATc1 was the one significantly upregulated when treated with rhein (Fig. 6B). To verify the possible interaction between rhein and NFATc1, molecular docking was applied and the score of combined energy was acquired (Table 2), indicating an ideal combination effect (< − 5 kcal/mol). In the complex NFATc1-rhein, the small molecule rhein bound to the NFATc1 protein in a pocket surrounded by amino acids VAL581, LEU487, GLN419, GLN580, LEU420, ALA582, ARG562, ASP484, where it formed hydrogen bonds with GLN580, LEU420, ALA582, ARG562, ASP484, and hydrophobic interactions with VAL581, LEU487, GLN419 (Fig. 6C). The local combination view is displayed in Fig. 6D, with the yellow dotted line indicating the hydrogen bonds, the green line demonstrating the amino acids forming hydrogen bonds with rhein, the blue figure displaying the corresponding protein, and the purple sticks showing rhein. To clarify whether rhein interacted with NFATc1, a biotin pull-down assay was carried out with the applications of biotinylated rhein (bio-rhein), with its chemical structure displayed in Fig. 6E. The results of western immunoblotting indicated the direct interaction between rhein and NFATc1 (Fig. 6F). To verify whether the regulatory role existed between NFATc1 and Trem2, the expression level of Trem2 was evaluated by RT-qPCR in NFATc1-siRNA transfected RAW264.7 cells, which showed that Trem2 expression was significantly decreased (Fig. 6G). To verify whether NFATc1 could potentially regulate the transcription of Trem2, the JASPAR database was applied. With the motif sequence (Fig. 6H), the screening revealed a TGGAAA binding site at 423 bp upstream of the transcription start site (TSS) of the Trem2 gene set (Fig. 6I). To identify the binding of NFATc1 in the Trem2 promoter region, a ChIP assay was performed in V5 (mock) and NFATc1-V5 expressing RAW264.7 cells using V5 antibody. The expression of NFATc1 associated with the binding site was ~ 5.8-fold higher as compared with the mock (Fig. 6J). To further confirm the target sequence of NFATc1 on the Trem2 promoter, luciferase reporter vectors were constructed with the insertion of the Trem2 promoter or a Trem2 promoter mutated by altering the nucleotide sequence from TGGAAA into TGGGGG (Fig. 6K). RAW264.7 cells were transfected with the NFATc1 expressing vector, followed by promoter activities evaluation via luciferase reporter assay. As compared with the group for the Trem2-wt reporter, the use of a triple-base mutation of the Trem2 promoter region resulted in a significant decrease of transcriptional activity. (Fig. 6K). In addition, luciferase assay was also carried out with the introduction of si-NFATc1 and si-NC. In the si-NC group, the administration of rhein significantly promoted the expression of Trem2 luciferase. By contrast, the Trem2 luc signal was significantly weakened and did not show a rhein-dependent stimulus changes in the si-NFATc1 group (Fig. 6N). DHE assay and immunofluorescent staining showed that the intracellular ROS level, CD86 and Trem2 signal in si-NFATc1 group did not display a significant change even with rhein pretreatment. By contrast, all the parameters were significantly downregulated in the control group with rhein administration (Fig. 6L, M, O–Q). Collectively, rhein executed its anti-inflammatory effects by interacting with NFATc1, which was the upstream TF of Trem2.

**Table 2:** Small molecule-protein binding affinity evaluation based on AutoDock Vina (kcal/mol)

Target name	Ligand name	Docking score
NFATc1	Rhein	− 6.8

### Blockage of NFATc1 in vivo blunted the inflammatory regulatory role of rhein in the ARDS model

**Fig. 7:**
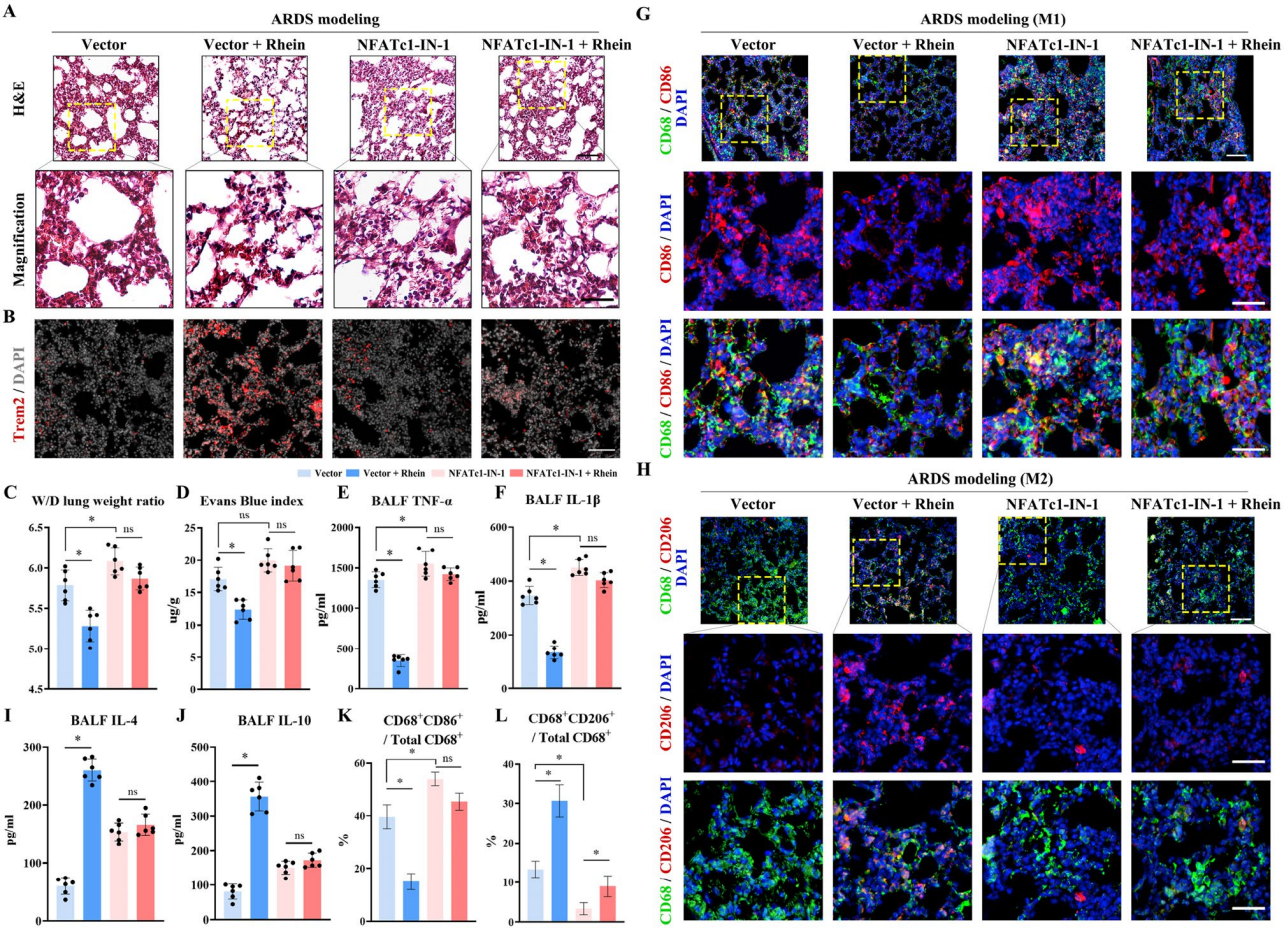
Blockage of NFATc1 alleviated the anti-inflammatory role of rhein in ALI/ARDS in vivo by downregulating Trem2 expression, followed by decreased macrophage M2 polarization transition. **A** Representative images of H&E stained lung tissues with zoomed views below (upper scale bar = 100 μm; lower scale bar = 50 μm; yellow dotted box indicating the zoomed area; *n* = 6 per group). **B** Representative images of immunofluorescence staining labeling via Trem2 (red) and nuclei (DAPI, blue) (scale bar = 100 μm; *n* = 6 per group) **C** Wet-to-dry (W/D) lung weight ratio (*n* = 6 per group). **D** Evans blue index (*n* = 6 per group). **E, F** Concentrations of TNF-α and IL-1β in the bronchoalveolar lavage fluid (BALF) (*n* = 6 per group). **G** Representative images of immunofluorescence staining labeling M1 macrophages via CD68 (green), CD86 (red), and nuclei (DAPI, blue) (upper scale bar = 100 μm; lower scale bar = 50 μm; *n* = 6 per group). **H** Representative images of immunofluorescence staining labeling M2 macrophages via CD68 (green), CD206 (red), and nuclei (DAPI, blue) (upper scale bar = 100 μm; lower scale bar = 50 μm; *n* = 6 per group). **I, J** Concentrations of IL-4 and IL-10 in the bronchoalveolar lavage fluid (BALF) (*n* = 6 per group). **K, L** Statistics of the proportion of M1 and M2 macrophages, indicated by CD68^+^CD86^+^ cells and CD68^+^CD206^+^ cells versus total CD68^+^ cells (*n* = 6 per group). *P < 0.05. means ± SD (Color figure online)

To clarify whether rhein played its regulatory role via the NFATc1/Trem2 axis in vivo, the ARDS model was established with the administration of an NFATc1 blocker (NFATc1-IN-1). The blockage of NFATc1 significantly attenuated the anti-inflammation effects of rhein, along with the upregulating expression of Trem2 on the ARDS model (Fig. 7A, B). The parameters of lung inflammation and pro-inflammatory cytokines, including wet/dry lung weight ratio, Evans blue index and TNF-α, IL-1β concentrations in BALF changed in a similar pattern (Fig. 7C–F). Via IF staining, it was observed that the inflammation inhibitory role of rhein and the function of promoting macrophage M2 polarization transition were blunted with treatment by NFATc1-IN-1 (Fig. 7G, H, K, L). The concentrations of macrophage M2 polarization-related markers, IL-4 and IL-10, were measured releasing in a similar pattern (Fig. 7I, J). The above findings proved that rhein executed its immunomodulation function through the NFATc1/Trem2 axis via interacting with NFATc1 directly.

### Schematic study diagram

**Fig. 8:**
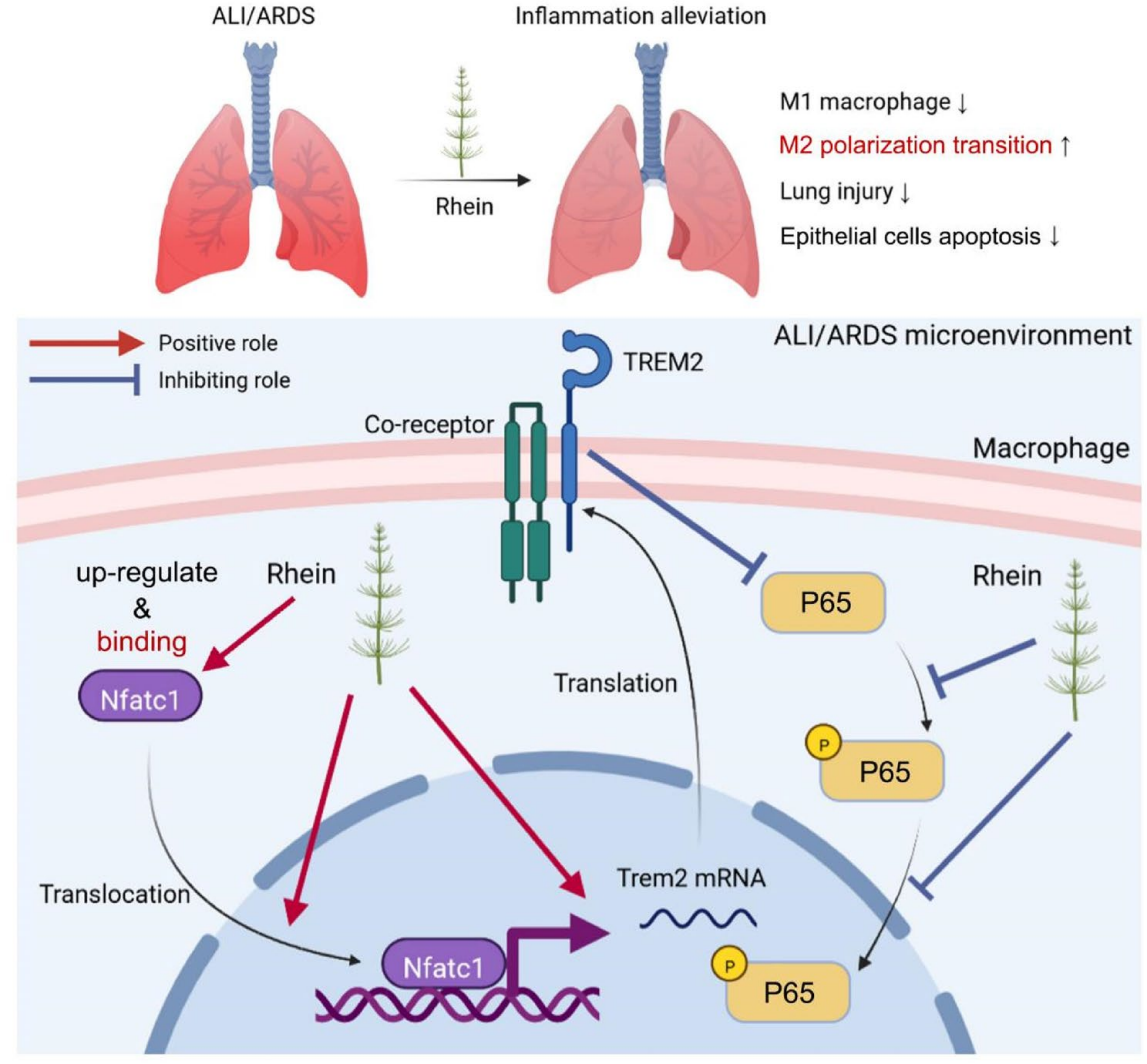
Schematic diagram of the study. The upper part of the diagram indicates the overall effect of rhein administration in ALI/ARDS, including decreasing M1 macrophages, upregulated M2 polarization transition, as well as alleviated lung injury and the epithelial cells apoptosis. The lower part of the diagram demonstrates the role rhein played with macrophages, including interacting with NFATc1 and activating the NFATc1/Trem2 axis, to promote the M2 polarization transition under ALI/ARDS microenvironment

The upper part of the diagram depicted the overall changes of ALI/ARDS with the treatment of rhein, while the lower part demonstrated the regulatory means of rhein at the intracellular molecular level (Fig. 8).

## Discussion

This study explored the role of rhein in ALI/ARDS induced by LPS in vivo and co-culture simulation in vitro. Our study showed that rhein could alleviate inflammatory infiltration after ALI/ARDS in vivo by reducing the number of M1 macrophages and improving M2 polarization transition of macrophages. In vitro, rhein could inhibit the effect of conditioned medium on the activation and translocation of NF-κB, at the intracellular ROS level, M1 polarization degree, and the expression levels of pro-inflammatory cytokines. In terms of the mechanism, combined with the transcriptome sequencing, the gene knockout mice model, molecular docking, biotin pull-down assay and ChIP assay, our study indicated that rhein promoted Trem2 expression by activating and interacting with NFATc1, the upstream transcription factor of Trem2. The upregulated Trem2 level inhibited the translocation and activation of NF-κB and finally suppressed the M1 polarization of macrophages, thus reducing the pulmonary inflammation after ALI/ARDS. In summary, rhein could be applied as a potential therapeutic plan for ALI/ARDS by activating the NFATc1/Trem2 signaling axis.

The pathophysiological mechanism of ALI/ARDS is closely related to the immune cells, with pulmonary macrophages playing the most important role in innate immunity [38, 39]. In the pathological process of ALI/ARDS, macrophages not only positively regulate the inflammatory response, but also actively take part in the repair of damaged tissue [40]. As highly plastic cells, macrophages can change their polarization state by receiving stimulus signals derived from the surrounding microenvironment, thus showing activated (M1) or regulated (M2) types. Previous studies have reported that in intestinal inflammation and gouty inflammation induced by uric acid crystals, rhein inhibited macrophage M1 polarization by inhibiting the activation of NLRP3 inflammasomes [16, 41]. Similarly, in this study, it was also observed that rhein decreased the intracellular ROS level, and inhibited the activation and translocation of NF-κB, thereby curbing the M1 polarization of macrophages in both in vivo or in vitro simulation of ALI/ARDS. In addition, it is worth mentioning that the LPS-treated alveolar epithelial cell supernatant was applied to stimulate the RAW264.7 cells, so as to simulate the microenvironment of ALI/ARDS in an accurate way.

The triggering receptor expressed on myeloid cells-2 (Trem2) is a signal hub that mediates immune response, which is widely distributed on the surface of myeloid cells [42–44]. In this study, we reported for the first time that rhein could upregulate the expression level of Trem2. In vivo, our study indicated that Trem2 was the downstream functional molecule for rhein and clarified the important prognostic impact of Trem2 on ALI/ARDS at the same time, through the applications of Trem2 knockout mice models, which were similar to the previous findings [45]. Trem2 mediates phagocytosis and inflammation [46–48], and regulates fibrosis and lipid metabolism [49–52]. In terms of structure, Trem2 lacks the intracellular signal peptide, so it displays co-receptor dependence when initiating intracellular signal transduction [53]. Through the interactions with different co-receptors and ligands, it can even attain the opposite downstream signal effect [54]. Taking NF-κB as an example, Trem2 played a significant promotive role in regulating the intracellular ROS level, NF-kB phosphorylation, and nuclear translocation in periodontitis-activated osteoclasts [33]. The upregulated Trem2 level was able to inhibit the activation of NF-kB and induce macrophages M2 polarized in microglia and tumor-associated macrophages [55–58]. In this study, it was observed that rhein-mediated upregulation of Trem2 expression significantly inhibited NF-kB activity and promoted the M2 polarization changes of macrophages in ALI/ARDS model.

Through the joint applications of target prediction and RNA sequencing, two transcription factors were screened out: NFATc1 and NF-κB. The nuclear factor of activated T cells cycloplasmic 1 (NFATc1), which belongs to the transcription factor family of nuclear factor of activated T cells (Nfat), is a pivotal regulatory molecule to the maturation and differentiation of osteoclasts [59, 60]. In addition to the classical function reported in the osteoclastogenesis, it was recently demonstrated that NFATc1 could also regulate the M2 polarization of macrophages [61]. In our study, the results of biotin pull-down assay indicated that rhein could directly interact with NFATc1. Further, ChIP assay and the dual luciferase reporter assay indicated that NFATc1 could bind the promoter region of Trem2 and initiate the transcription. Interestingly, the transcriptional activity of NFATc1 was positively correlated with the application of rhein in the system, which further revealed that rhein could upregulate the expression of Trem2 by activating NFATc1.

The nuclear factor κB (NF-κB) has long been considered as a typical pro-inflammatory pathway, which was mainly based on its pro-inflammatory downstream factors, including cytokines and chemokines [62]. A large number of previous studies proved that rhein could directly inhibit the activity of NF-κB [63, 64]. Interestingly, in our study, the elimination of Trem2 or NFATc1 remarkably mitigated the beneficial effects of rhein applications in the ALI/ARDS model, suggesting that rhein mainly hindered the M1 polarization of macrophages and promoted the M2 polarization transition by interfering with the upstream signal (NFATc1) of Trem2 rather than the downstream signal (NF-κB), thus regulating the prognosis of ALI/ARDS.

Although this study fully demonstrated the application of rhein on the pathophysiological changes of LPS-induced ALI/ARDS and its mechanism for the first time, several concerns need yet to be addressed: 1) Whether the administration pathways will affect the prognosis and outcome of ALI/ARDS when rhein is applied; 2) In the ALI/ARDS model, why does rhein affect the polarization of macrophages and the outcome of ALI/ARDS mainly by interfering with the upstream signal of Trem2 (NFATc1) rather than the downstream signal (NF-κB)?

Collectively, this work provides the important evidence that rhein inhibits lung tissue inflammatory responses and promoted M2 polarization transition via activating the NFATc1/Trem2 signal in LPS-induced ARDS, which shed more possibilities on the clinical treatments of this pathological process.

## Data Availability

All data generated in this study will be provided by the corresponding author upon request.
